# A Multi-faceted Educational Approach for Pain Metric Recording Prior to Knee and Hip Arthroplasty: Effects on Documentation by an Acute Pain Service

**DOI:** 10.7759/cureus.5030

**Published:** 2019-06-28

**Authors:** Alberto Ardon, Matthew Warrick, Tyler Wickas

**Affiliations:** 1 Anesthesiology, Mayo Clinic, Jacksonville, USA; 2 Anesthesiology, University of Florida, Jacksonville, USA; 3 Anesthesiology, Florida State University, Tallahassee, USA

**Keywords:** education, electronic prompt, analgesic documentation, acute pain service, multimodal analgesia, arthroplasty

## Abstract

Background

Despite the increased use of electronic medical records (EMRs) in past years, the recording of clinically useful baseline pain information may still be lacking. An educational effort targeted at the acute pain service and reinforced by electronic prompting may be an effective way to promote electronic documentation of relevant pain metrics. The objective of this study was to assess whether an educational effort with electronic prompting in the EMR promotes the documentation of baseline pain scores and preoperative opioid use by an acute pain service (APS).

Methods

A total of 98 patients were included in this study: 49 in the study group and 49 in the control group. The study group consisted of patients who underwent knee and hip arthroplasties after the institution of a multimodal analgesia educational program that also incorporated an electronic prompt to promote behavior change. Primary outcomes were the frequency of documentation of baseline pain scores and preoperative opioid use.

Results

After the implementation of the education initiative, 67% of the patients had baseline pain scores recorded in the preoperative APS documentation, compared to 20% in the control group (*p *= 0.0001). Preoperative opioid use was recorded in 24% of APS documentation within the control group, but this increased to 73% after the educational intervention (*p *= 0.0001). Documentation of resting pain scores on the day of surgery also increased from 59% to 87% (*p *= 0.0014).

Conclusions

The introduction of a multi-dimensional educational effort focused on baseline pain metric recording within the context of an analgesic change of practice increased assessment of both baseline pain and preoperative opioid use by APS. These results can be applied to other settings in which a focused change of practice is required and an electronic medical record already utilized.

## Introduction

The presence of electronic medical records (EMRs) has become almost ubiquitous over the course of the past decade. Specific to perioperative medicine, EMRs have become integral to accurate documentation of patient vital signs and medication administration, as well as capable data repositories and tools to investigate perioperative outcomes. Despite these advantages, an EMR system is only as useful as the data contained therein and the initial input of a patient’s relevant health variables is critical. Several recent investigations suggest that prompts in the EMR encouraging healthcare providers to record a particular data point may be influential on behavior and may even have a role in the accuracy and reducing documentation errors [1-4]. 

Within the realm of perioperative pain management, the accurate recording of analgesic metrics such as pain scores, functional status, and opioid use is critical to providing personalized and effective analgesic interventions. In the context of postoperative analgesia, an important data point that is sometimes overlooked or not recorded in preoperative documentation is a patient’s baseline pain score. Some evidence suggests that the higher the patient’s preoperative pain level, the more likely the patient will have postoperative pain that will be difficult to control or will develop chronic pain [5-7]. Furthermore, for extremity surgery such as arthroplasty, this baseline pain score should be specific to the operative site to contextualize postoperative pain scores and should include both static and active parameters. Likewise, qualitative and quantitative knowledge of a patient’s preoperative opioid analgesic use, if any, may influence the planning and efficacy of postoperative analgesic efforts [8-10]. Furthermore, if this information is recorded prior to surgery instead of after, the risk of recall bias (error caused by the inaccurate recollection of information by study participants) is decreased [11-12]. Recording of this surgery-specific baseline pain data by anesthesia providers in a consistent manner may be challenging. At our institution, approximately less than 25% of elective arthroplasty patients had this information recorded in the EMR. However, an educational effort targeted at involved providers and reinforced by electronic prompting may be an effective way to promote electronic documentation of these metrics. Such an educational program can be provided as part of the implementation of a multimodal analgesic practice guideline for hip and knee arthroplasties. The primary objective of this study is to assess whether a multi-faceted educational effort promotes the documentation of baseline pain scores and preoperative opioid use by an acute pain service (APS).

## Materials and methods

The University of Florida - Jacksonville Institutional Review Board approved this study. In this retrospective chart review, the two study groups were:

· Intervention group: consisted of patients undergoing total hip or knee arthroplasties after August 3, 2015, the first surgical date after the educational effort and implementation of the multimodal analgesic guideline.

· Control group: an equivalent cohort matched for age, American Society of Anesthesiologists (ASA) classification, and surgery type who underwent hip or knee arthroplasty prior to August 3, 2015.

To increase the homogeny between the two study groups, exclusion criteria for initial review were age less than 18 years, bilateral surgery, fracture as preoperative surgical diagnosis, placement of antibiotic spacer, and preoperative patient refusal for regional anesthesia.

A total of 152 patient charts were reviewed for this study. In total, 54 patients were excluded secondary to administration of one or more of the non-opioid analgesics (in the control group), clinical contraindication to one or more of the non-opioid analgesics or the peripheral nerve block. A total of 98 patients were therefore included in this study. A chart review consisted of recording the following information: age, gender, American Society of Anesthesiologists (ASA) score, site of surgery, whether primary or revision surgery, documentation of preoperative baseline pain score and opioid use, regional anesthetic technique employed, administration of acetaminophen, celecoxib, and gabapentin, and postoperative static and active pain scores.

Pre-implementation education

**Figure 1:**
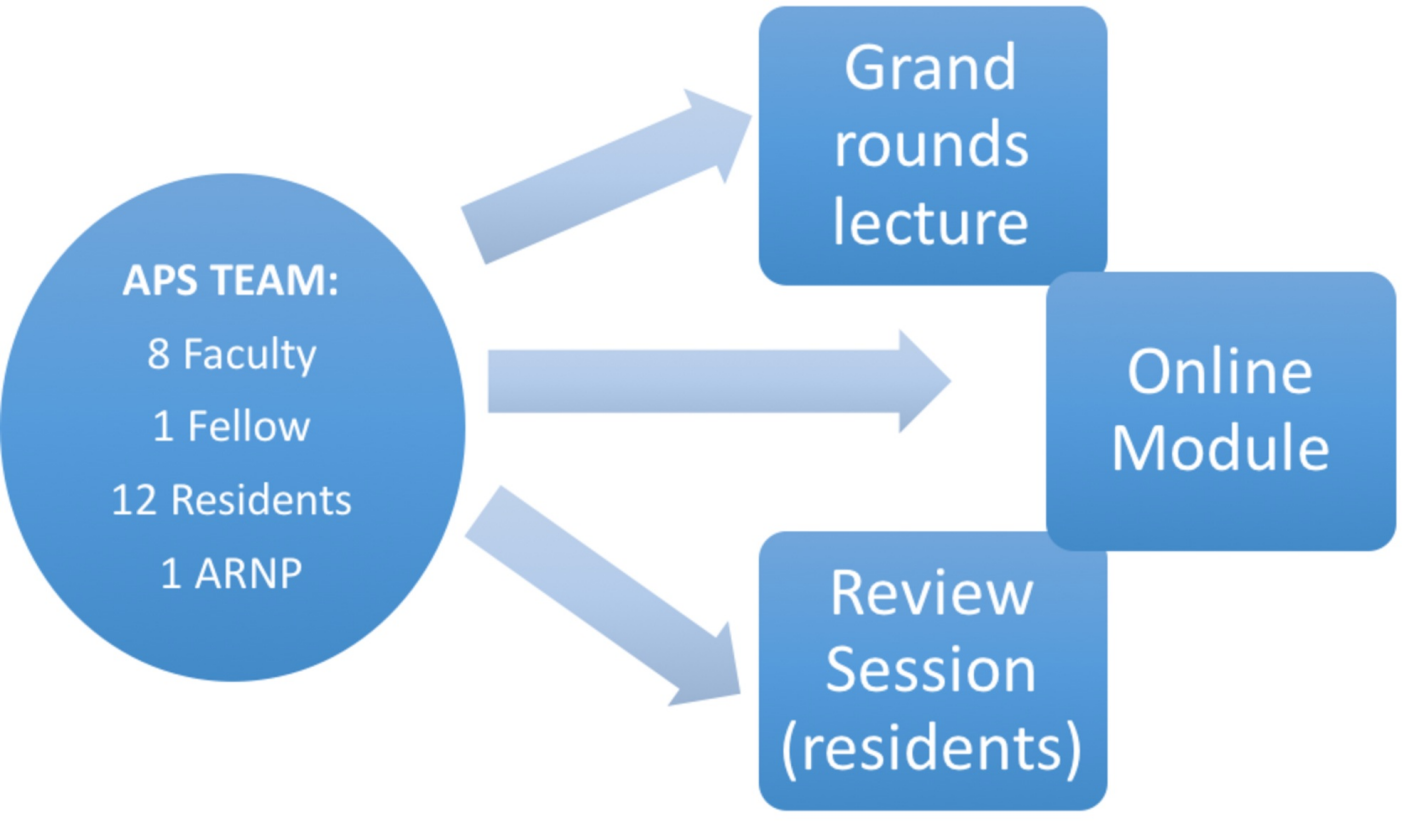
Pre-implementation education program components and target audiences APS, acute pain service; ARNP, advanced registered nurse practictioner

As a component of the multimodal arthroplasty guideline, all clinical members of the anesthesia APS (eight faculty, one fellow, 12 residents, and one acute pain nurse practitioner) participated in a pre-implementation educational program. With regard to the current study, the purpose of this educational effort was to introduce and reinforce the concept and benefits of multimodal analgesia and more surgery-specific pain data recording. Specific aims with regards to the current study were to 1) promote a more thorough preoperative review of patient pain history, 2) encourage documentation of preoperative baseline pain scores and opioid use, and 3) reinforce postoperative pain score recording. The educational effort consisted of three sessions: one grand rounds lecture, one online module, and one separate review session with residents, who would be the primary individuals evaluating patients and documenting outcome metrics (Figure 1).

Multimodal guideline and peri-operative documentation in electronic medical record

**Figure 2:**
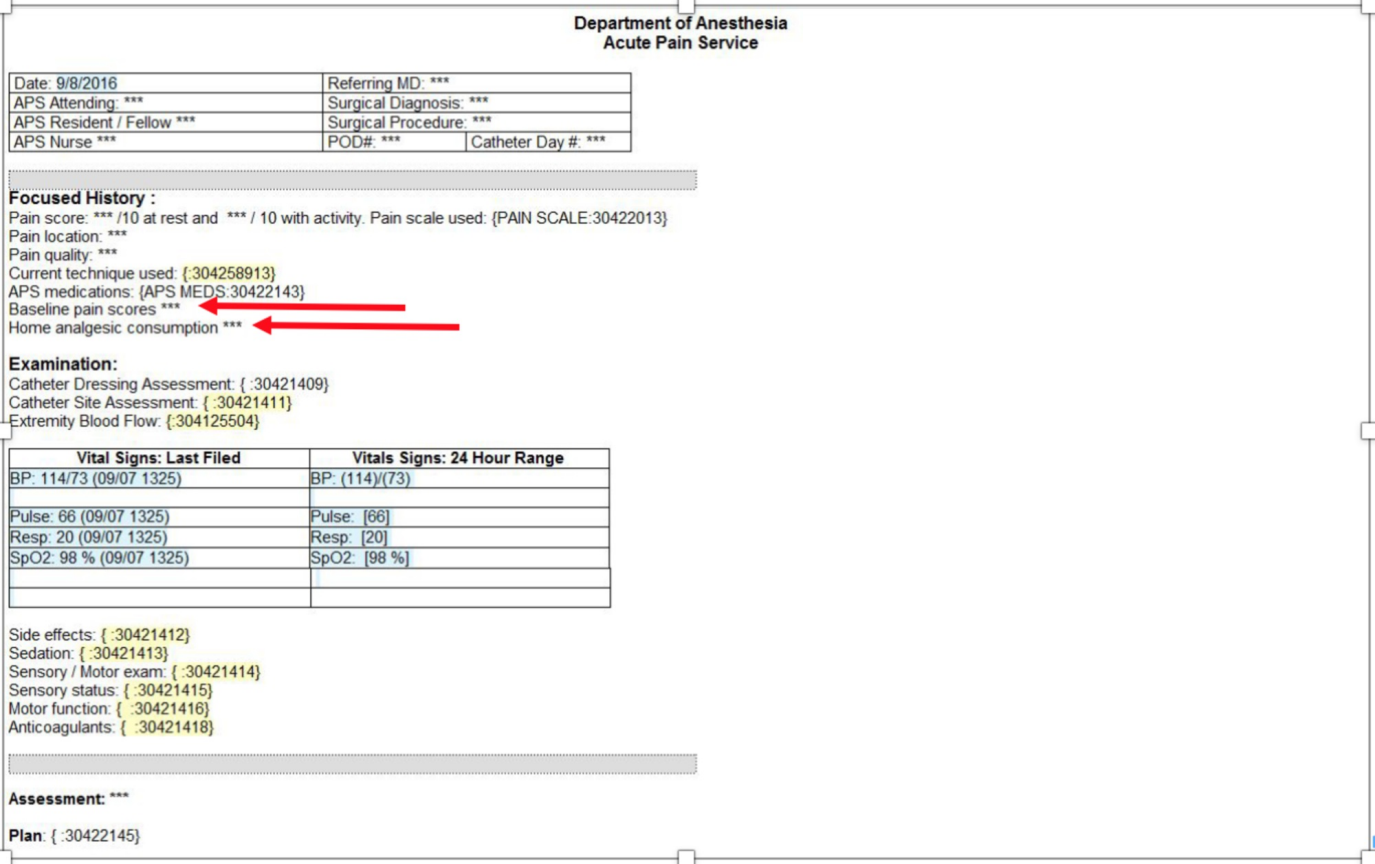
Acute pain service consult note Arrows point to electronically prompted sections to document baseline pain scores and opioid use. APS, acute pain service; POD, postoperative day; MD, medical doctor; BP, blood pressure; SpO_2_, pulse oximetry

With the introduction of the multimodal analgesic guideline in August 2015 (Table 1), the electronic documentation as filled by the APS was updated to provide electronic prompts for baseline pain scores and opioid use (Figure 2).

**Table 1:** TKA and THA multimodal analgesic components POD indicated as needed POD, postoperative day; TKA, total knee arthroplasty; THA, total hip arthroplasty

Preoperative Phase	Medications	Dose	Frequency
	Acetaminophen	1000 mg oral	Once
	Celecoxib	400 mg oral	Once
	Gabapentin	600 mg oral	Once
	Regional analgesia		
	Femoral (TKA) or Lumbar Plexus (THA) nerve block	25-30 ml ropivacaine	
Postoperative Phase	Medications	Dose	Frequency
	Acetaminophen	1000 mg oral	Every 6 hours for 48 hours
	Celecoxib	200 mg oral	Every 12 hours for 48 hours
	Gabapentin	600 mg oral	Every 12 hours x 2 doses, then every 8 hours for 24 hours
	Regional analgesia		
	Femoral (TKA) or lumbar plexus (THA) nerve catheter	Bupivacaine 0.125% at 10-12 ml/hr	Continuous until the morning of POD2

During the establishment of this change in the documentation, practitioners were encouraged to record 1) pain scores at the surgical site (at rest and with motion), 2) ambulatory opioid analgesics, if any, and 3) dose and frequency of those analgesics if applicable. These variables were obtained by directly asking patients the following questions:

· On a scale from 0 to 10 (0 being no pain and 10 being worst pain imaginable), how much pain do you currently have in your operative knee/hip at rest? With motion?

· During the past week, what opioid medications have you taken for pain on a daily basis?

· What dose of that opioid do you take and how many times a day?

Outcome measures

Primary outcomes were the frequency of assessment of 1) preoperative pain scores and 2) preoperative ambulatory opioid use as documented in the APS consult note. Preoperative opioid use was defined as a patient taking oral morphine, oxycodone, hydromorphone, methadone, or hydrocodone daily for the week prior to surgery, or a fentanyl transdermal patch for at least one week prior to surgery. Secondary outcomes included frequency of documentation of actual intake of preoperative opioid (usual total dose in a 24-hour period), and frequency of recording of postoperative pain scores (at rest and with motion) by APS on surgical follow-up. Administrations of non-opioid analgesics and peripheral nerve catheter use were also assessed. The use of these multimodal analgesic approaches was considered as consistent with the program guideline if the patient received a peripheral nerve catheter and all three non-opioid analgesics in the preoperative bay and these analgesic methods were continued on postoperative day (POD) 1.

Statistical analysis

Based on an estimated rate of baseline pain score and preoperative opioid use assessments of approximately 20% given current clinical practices prior to the establishment of the multimodal approach, we estimated that to detect a 30% change in incidence (with an alpha of 0.05 and Power of 80%), at least 38 patients would be required in each group. Statistical analysis was conducted using AcaStat (AcaStat Software, Poinciana, FL). Two-tailed unpaired *t*-tests were used for quantitative variables; *χ*^2^ analysis was utilized for nominal variables.

## Results

Baseline patient characteristics are shown in Table 2. No statistically significant differences existed regarding age, gender, ASA score, or surgery type. Approximately 88% of patients in the study group underwent a primary arthroplasty, compared to 69% in the control group (*p *= 0.027).

**Table 2:** Baseline characteristics *P* value < 0.05 denoted by asterisk (*). ASA, American Society of Anesthesiologists; TKA, total knee arthroplasty; THA, total hip arthroplasty

		Control (n = 49)	Study (n = 49)	P value
Age		57.4 (10.1)	61(9.5)	0.07
Gender	Male	47%	35%	0.22
	Female	53%	65%	
ASA (median)		3	3	0.45
Surgery	TKA	67%	61%	0.53
	THA	33%	39%	
Primary or revision*	Primary	69%	88%	0.027
	Revision	31%	12%	

Primary outcomes

Primary outcomes are shown in Table 3. Approximately 20% of patients in the control group had baseline pain scores recorded in the preoperative APS documentation, compared to 67% in the study group (*p *= 0.0001). Of those patients whose baseline pain score was assessed, there was no significant difference in the baseline pain score before (7.70 ± 1.89) and after (7.52 ± 1.54) multimodal guideline implementation (*p *= 0.75). Preoperative opioid use was recorded in 24% of APS documentation within the control group, but this percentage increased to 73% after the educational intervention (*p* = 0.0001).

**Table 3:** Primary and secondary outcomes *P* value < 0.05 denoted by asterisk (*); APS, acute pain service; POD, postoperative day

		Control (n = 49)	Study(n = 49)	P value
Baseline pain score assessed?*	Yes	20%	67%	0.0001
Baseline pain score (if available)		7.70 (1.89)	7.52 (1.54)	0.75
Preoperative opioid use assessed?*	Yes	24%	73%	0.0001
Preoperative opioid use (if available)	Yes	83%	69%	0.35
Among those whose pre-op opioid use was assessed as “yes”, what percentage had actual opioid intake documented?*		68%	20%	0.0001
Static pain score recorded by APS*	POD 0	59.2%	87%	0.0014
	POD 1	79.6%	91.8%	0.08
Active pain score recorded by APS	POD 0	28.6%	18.4%	0.23
	POD 1	59.2%	40.8%	0.225
Use of non-opioid analgesics	Acetaminophen		100%	
	Celecoxib		88%	
	Gabapentin		98%	
Regional analgesic technique	Catheter	82%	92%	0.09
	Single shot	8%	6%	
	Intrathecal opioid	0	2%	
	Failed block	10%	0	

Secondary outcomes

Secondary outcomes are also shown in Table 3. Of the patients who did have preoperative opioid use assessed, 83% of control group patients used preoperative opioids versus 69% of study patients (*p *= 0.35). Among these patients, 68% of control patients had 24-hour preoperative opioid intake documented, compared to only 20% of study patients (p=0.0001). Regarding documentation of POD 0 and POD 1 resting and active pain scores by APS, only documentation of resting pain scores on POD 0 resulted in a statistically significant difference between the two groups (59% vs 87%, *p* = 0.0014). Use of multimodal analgesics was 100% for acetaminophen, 88% for celecoxib, and 98% for gabapentin after the introduction of the analgesic guidelines. Peripheral nerve catheter use (rather than single shot nerve blockade) did not increase significantly (82% vs 92%, *p* = 0.09). Approximately 10% of patients in the control group were found to have no sensory deficit in the recovery area and thus were classified as “failed block”.

As more patients in the protocol group underwent primary arthroplasty, outcomes were re-examined within this sub-population (Table 4). Rates of baseline pain score and opioid use assessment were higher for study patients (24% vs 67%, *p *= 0.0001; 30% vs 74%, *p *= 0.0004, respectively), consistent with general results.

**Table 4:** Primary outcome measures controlling for primary arthroplasty patients *P* value <0.05 denoted by asterisk (*)

		Control (n = 34)	Study (n = 43)	P value
Baseline Pain Score Assessed?*	Yes	24%	67%	0.0001
Baseline pain score (if available)		7.25 (1.83)	7.52 (1.55)	0.68
Preoperative opioid use assessed?*	Yes	30%	74%	0.0004

## Discussion

In our study, the use of a multifaceted educational program that included didactic instruction and electronic prompting emphasizing documentation of baseline pain characteristics in the EMR was associated with a significant increase in recording of both baseline pain scores and opioid use. This finding is in agreement with previous research which suggests that both educational efforts and electronic prompting are effective in causing behavior change [1-4,13]. It is also in agreement with studies that suggest that behavior change is more likely when a practical application is included within the educational framework [14-16]. To our knowledge, however, this is the first study to demonstrate these effects within the context of an APS. Clinical educational efforts may have an ultimate higher rate of success when adapted to the local clinical framework and accompanied by active reinforcement such as simulation or teaching [17-19]. Clinical guidelines, which encourage target activities and promote practices learned in above educational efforts, may be advantageous to translate knowledge gain into internalized behavior change. While documentation of baseline pain or opioid use was encouraged in this project via education and facilitated electronically, it was not made compulsory so as to not introduce bias. In other words, the APS consult note could be completed without this baseline pain information. Thus, clinicians who, after the introduction of this change in the documentation, recorded the information did so after a visual electronic prompt which could have been bypassed if wanted. Thus, we believe that the observed increase in frequency in the documentation of baseline pain scores and preoperative opioid use was indeed secondary to the intervention. The implication of this work is that a simple educational intervention, coupled with a behavior change prompt, can alter clinician behavior and promote better documentation. Although our study examined this behavior change purely within the framework of an APS, the concept could be applied across different clinical contexts and learner populations.

With regards to our patient population, the establishment of this multimodal analgesic guideline: 1) revealed that patients undergoing arthroplasties at our institution had a fairly high baseline pain score (>7/10), and 2) suggest that almost three-quarters of our hip and knee arthroplasty patient population may use opioid medication on a regular basis prior to surgery. Knowledge of these high preoperative pain scores and rates of use of opioid analgesics is very useful for the analgesic management of patients after major surgery such as arthroplasty for two reasons. First, the accurate assessment of preoperative pain scores provides a relative framework within which to build both clinician and patient expectations, which can affect post-surgical outcome [12]. A patient who, for example, has a numeric baseline knee pain score of 8 out of 10 should be counseled that they may be at increased risk of postoperative pain that may be more difficult to control. Second, identification of patients who have significant recent or current exposure to opioids prior to surgery allows the clinician to assess the risk of opioid tolerance and adjust the anesthetic and analgesic plan accordingly, given recent evidence that exposure to opioids may indeed alter anesthetic/analgesic requirements [20].

Despite the improvement in recording the baseline preoperative opioid use, the recording of the actual amount of preoperative opioid analgesic consumed by patients in a 24-hour period did not improve and in fact worsened after the educational intervention (68% vs 20%, *p *< 0.001). The reasons for this decrease in recording of this information are unclear but may be secondary to 1) a significantly smaller number of patients (*n *= 10) that were confirmed to be using opioids preoperatively in the control group compared to those in the intervention group (*n *= 25); 2) a need for further, more targeted educational reinforcement for obtaining this particular parameter or 3) an overall need for deeper cultural change with respect to the significance of this data. We believe that obtaining a relative idea of the amount of opioid, if any, that a patient is consuming prior to undergoing a major operation is important as this variable can influence the patient’s postoperative risk profile as well as directly affect intraoperative and postoperative analgesia.

Although the recording of static pain scores by APS on POD 0 increased by 28%, the recording of active pain scores did not change significantly on either POD 0 or POD 1. Differentiating between static and active pain may be critical in establishing appropriate analgesia to aid in physical therapy. Thus, it is concerning that this documentation did not increase. However, in retrospect, the recording of these specific parameters could have been addressed more thoroughly in the educational curriculum. More education may be required, as clinician biases may influence behavior and decrease the impact of clinical documentation changes [21]. Consequently, educational materials at our institution now include an emphasis on differentiating and recording both static and active pain, which may also be applicable to other institutions as the use of active/functional pain scores increases in clinical practice overall.

Limitations

Some limitations exist in our study. First, despite an increase in the documentation of whether or not a patient was taking preoperative analgesics, documentation of the actual daily dosage prior to surgery was poor after implementation of the protocol. In fact, the rate of recording of this variable decreased by 48%. We suspect the poor recording of this variable suggests a need for either an increased educational emphasis on this variable or an electronic prompt that first asks if the patient has preoperative opioid use and, if the response is yes, thereafter asks the clinician document the 24-hour intake. Without knowledge of the extent of preoperative opioid exposure, assessing relative postoperative opioid requirements may be very difficult. Likewise, as tolerance is defined as the use of 60mg of oral morphine equivalents per day for at least seven days whether the patient is actually opioid tolerant or the degree to which the patient is at risk for opioid-related side effects immediately postoperatively can be unknown or at the very least subject to recall bias if this information is not obtained prior to surgery [22]. Indeed, some research suggests that opioid-tolerant patients are in fact at increased risk of respiratory depression compared to an opioid-naïve population [23].

Second, an issue that deserves consideration is whether the observed changes in behavior were secondary to factors outside the boundaries of the study. Specifically, 1) had clinicians been exposed to any other educational interventions on the same topic previously? and 2) did social pressure rather than the intervention cause a behavior change? For the first point, no other concurrent educational intervention or hospital policy was implemented or ongoing six months prior to or during the implementation of our educational/clinical protocol. While it may be possible that some clinicians had previously had some education on this topic, the use of multimodal analgesia and a comprehensive peri-operative pain documentation approach was not commonplace in our practice, and thus any previous exposure is unlikely to have influenced the observed results. Regarding social pressure, while this is certainly a possibility, we believe that such social pressure would arise as a consequence of the study intervention, and thus still be attributed to the educational/electronic prompting system. Therefore, we believe that overall the change in behavior as indicated by an increase in the documentation was, in fact, a result of the efforts described in this study.

Lastly, the reason for a 10% incidence of "failed block" in the control group (compared to 0% in the study group) is unclear. We speculate that the decrease in block failure after the study intervention was related to an educational component that encouraged assessing sensory deficit soon after peripheral nerve blockade, but this explanation assumes that no other change in clinical practice occurred.

## Conclusions

In conclusion, the introduction of a multi-dimensional educational effort for the implementation of a multimodal analgesic guideline increased assessment of both baseline pain and preoperative opioid use by the acute pain service. These results can be applied to other settings in which a focused change of practice is required and an electronic medical record already utilized. Future studies should be done to further examine and prospectively verify and supplement these results.
